# A comprehensive comparison study of three different planar IMRT QA techniques using MapCHECK 2

**DOI:** 10.1120/jacmp.v14i6.4398

**Published:** 2013-11-04

**Authors:** Vance P. Keeling, Salahuddin Ahmad, Hosang Jin

**Affiliations:** ^1^ Department of Radiation Oncology University of Oklahoma Health Sciences Center Oklahoma City OK USA

**Keywords:** IMRT quality assurance, MapCHECK, angular dependency, Varian IGRT treatment couch, field by field

## Abstract

The purpose of this study is to determine comparability of three different planar IMRT QA techniques: patient gantry angle composite (PGAC), single gantry angle composite (SGAC), and field by field (FBF), using MapCHECK 2 device and the γ test as performance metrics; and to assess the dependency of these techniques on intensity modulation, couch attenuation, and detector position (angular dependency). Ten highly modulated head and neck (H&N) and ten moderately modulated prostate IMRT validation plans were delivered using different techniques and were intercompared using the Student's t‐test. The IMRT QA measurements were evaluated by percentage of points passing the γ test for three different criteria: 1% (dose difference)/1 mm (distance to agreement (DTA)) (C1), 2%/2 mm (C2), and 3%/3 mm (C3). To investigate dependency of the IMRT validation on treatment couch, ionization chamber measurements, as well as the conventional MapCHECK 2 QAs, were performed with PGAC and PGAC‐WOC (without couch; using an extended tennis racket‐type insert with negligible attenuation assumed). To determine angular dependency of the MapCHECK 2, patient gantry field‐by‐field (PG‐FBF) technique was delivered and evaluated separately for each field. The differences of γ passing rates between SGAC and FBF were statistically insignificant, while these were statistically significant when compared to PGAC. SGAC and FBF techniques showed statistically insignificant differences between different levels of intensity modulation (H&N vs. Prostate) at C2 and C3 criteria, while PGAC could not for any criteria. The treatment couch has a significant impact on γ passing rates (PGAC vs. PGAC‐WOC), but an ionization chamber‐based IMRT validations showed clinically insignificant dose errors (< 2%) in all cases. This study showed that the MapCHECK 2 device has large angular dependency, especially at gantry angles of 90° and 270°, which dramatically affected the γ passing rates of PGAC. With proper consideration of couch attenuation and beam arrangement, the MapCHECK 2 will produce clinically comparable QA results using the three different planar IMRT QA techniques.

PACS numbers: 87.55.km, 87.55.Qr, 87.56.Fc

## I. INTRODUCTION

Intensity‐modulated radiation therapy (IMRT) has become a common treatment practice in nearly all radiation clinics. Since its inception, a large number of quality assurance (QA) methods have been developed to test the accuracy of treatments. One of the most widely used patient‐specific IMRT QA techniques is the 2D dosimetric comparison between planned and measured dose distributions. The 2D planar comparison can be accomplished using films or a 2D detector array with three different techniques: patient gantry angle composite (PGAC), single gantry angle composite (SGAC), and field by field (FBF). The PGAC technique is accomplished by delivering the entire treatment with the same beam geometry that is used for treatment plan. In the SGAC technique, the entire treatment is delivered with all fields at gantry angles of zero degree; and in the FBF technique, each treatment field is analyzed separately with all fields delivered at gantry angles of zero degree.

Assessment of these QA procedures in most institutions is determined by evaluating planned and measured dose distributions through the use of the γ test.^(1)^ The γ test is an evaluation metric that combines dose difference and distance‐to‐agreement (DTA) criteria. The dose difference criterion is useful in regions of low‐dose gradient, while the DTA criterion is more helpful in regions of high‐dose gradient. Unlike other QA methods (e.g., composite analysis^(2)^) indicating a result of pass or fail, the γ test gives a numerical value allowing the user to determine quantitatively if a point passed or failed. Most institutions are using γ criteria of 3% dose difference and 3 mm DTA for IMRT QA analysis.^(3)^ A research group^(4)^ recommended these specific criteria to produce meaningful and consistent QA results. The acceptable γ passing rates for prostate and other complicated treatments are found to be ≥95% and ≥90%, respectively, when using 3%/3 mm criteria.^(^ ^3^ ^,^ ^5^ ^)^ However, these criteria are based on what is achievable, and not on what is clinically acceptable.

Nelms and Simon^(6)^ showed that 32.8% of the institutions are using the SGAC method for 75%‐100% of their patients; and 64.1% of the institutions are using FBF technique for all of their patients. PGAC analysis has also been widely used for IMRT QA (e.g., planar film QA) and is recently becoming more popular due to intensity‐modulated arc therapy (IMAT).^(^ ^7^ ^,^ ^8^ ^)^ Even though these QA methods are widespread, there are very few extensive comparison studies on the relevance of the different techniques. The purpose of this study is to improve the understanding of these three different planar IMRT QA techniques (PGAC, SGAC, and FBF) using a diode array device MapCHECK 2, by comparing the γ passing rates as a performance metric. Also, the dependency of γ passing rates on level of intensity modulation, couch attenuation, and detector position (angular dependency) is studied.

## II. MATERIALS AND METHODS

### A. IMRT verification plans

IMRT verification plans were generated by the Varian Eclipse treatment planning system (TPS) version 8.9 (Varian Medical Systems, Palo Alto, CA) for ten H&N and ten prostate patients with step‐and‐shoot technique. The H&N treatment plans were more modulated than the prostate plans, and the level of complexity was demonstrated by number of monitor units and beam segments, as summarized in Table 1.

**Table 1:** Summary of the IMRT QA patients (split beams were individually counted in the number of beams)

	*Head and Neck*					*Prostate*			
*Patient No*.	*No. of Beams*	*Total MUs*	*No. of Segments*	*Energy*	*Treatment Site*	*No. of Beams*	*Total MUs*	*No. of Segments*	*Energy*
1	12	1087	192	6 MV	Nasopharynx	7	494	107	10 MV
2	12	879	140	6 MV	Pharynx	9	472	133	10 MV
3	9	583	130	10 MV	Esophagus	9	317	122	10 MV
4	9	1039	113	6 MV	Rt. Parotid	9	550	149	6 MV
5	7	420	107	6 MV	Esophagus	9	366	133	10 MV
6	18	1077	244	6 MV	Tongue	9	438	128	6 MV
7	12	808	188	6 MV	Larynx	7	572	108	6 MV
8	12	877	162	6 MV	Larynx	7	387	98	6 MV
9	14	810	159	6 MV	Tonsil	8	433	117	6 MV
10	10	775	188	6 MV	Tonsil	7	363	96	6 MV
Mean		836	162				439	119	
SD		213	42				84	17	

### B. IMRT QA delivery and devices

**Figure 1:**
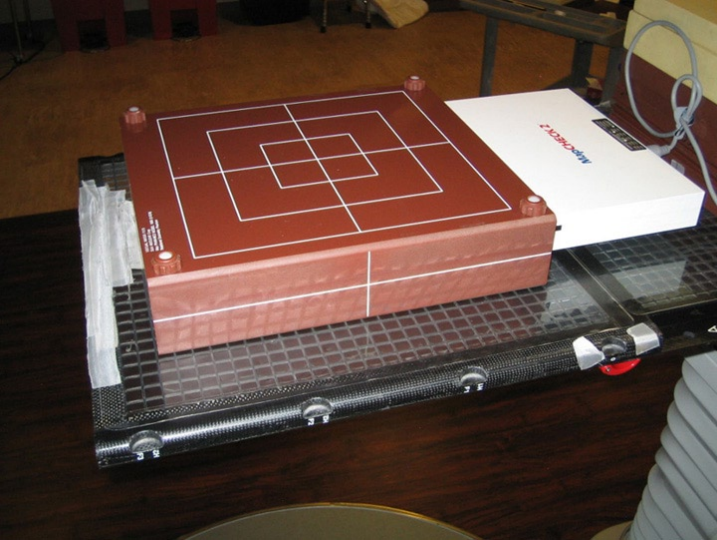
MapCHECK 2 device set up for measuring IMRT validation plans using extended tennis racket‐type grid insert.

All IMRT verification plans were delivered using the Varian TrueBeam STx (Varian Medical Systems) with high definition multileaf collimator (HD120 MLC; leaf width of 2.5 mm in the center region (32 leaf pairs) and 5.0 mm in the outer part (28 leaf pairs)). A report of IMRT H&N phantom irradiation from the Radiological Physics Center (RPC) using our TrueBeam system showed that the ratio of RPC value to our value was found to be less than 2% for planning target volume (PTV); and 0 mm of two dosimetry points in a spinal cord for organs at risk (OARs). The criteria for acceptance set by RPC (RPC value/institutional value) have been 7% for PTV and less than 4 mm for OAR. The accuracy of the 20 IMRT QA plan deliveries was verified using point dose measurements with an ionization chamber (PTW TN31010; PTW, Freiburg, Germany). A solid water phantom (30cm×30cm×10cm) was scanned using a GE LightSpeed 16 slice CT scanner (GE Healthcare, Waukesha, WI) and the images were transferred to the TPS for QA planning. The measurements were performed at isocenter or at a point of high‐dose and low gradient using an extended tennis racket‐type grid insert (see the couch top only in Fig. 1; negligible couch attenuation assumed) and compared to corresponding dose points in TPS calculations without the couch top. The ionization chamber measurement was converted to dose by normalizing it to a measurement in a calibration condition (1 cGy/MU at $D_{\text{max}}$ for a 10cm×10cm field and 100 cm SSD). Percent differences between the planned doses (P) and measured doses (M) were determined by (M−P)/P×100(%).

The Sun Nuclear Corporation (SNC) MapCHECK 2 (serial number: 6959303; Sun Nuclear Corporation, Melbourne, FL) with MapPHAN‐MC2 was used to measure the dose distributions using PGAC, SGAC, and FBF techniques. The MapCHECK 2 is a 2D array of 1527 n‐type diodes separated by a distance of 10.0 mm horizontally and vertically while being separated by 7.1 mm diagonally. The MapPHAN‐MC2 is a solid water block (34.9cm×37.9cm×8.0cm) with water‐equivalent buildup of 5.0 cm above and below the detector plane of MapCHECK 2 (Fig. 1).^(9)^ The MapCHECK 2 with MapPHAN‐MC2 was scanned using the GE CT scanner and transferred to the Eclipse TPS for dose calculations of IMRT QA plans on the phantom. The raw CT data of MapCHECK 2 were used because lateral beams that were significantly affected by CT artifacts were not used in this study, and to avoid dosimetric uncertainty caused by HU values overridden on CT artifacts.

### C. Compatibility of three different IMRT QA methods: PGAC, SGAC, and FBF

A number of institutions perform their IMRT QA without considering couch attenuation for patient planning as a common clinical practice.^(^ ^7^ ^,^ ^10^ ^)^ In order to simulate clinical situations, the IMRT QA delivery was performed with the Varian Exact IGRT couch top, while the plan dose calculations were done without considering the couch top. The effect of couch attenuation was separately investigated in this study (see Materials and Methods section D below). The MapCHECK 2 measurements for all three techniques were analyzed using the SNC patient software (version 6.0; Sun Nuclear Corporation). The γ criteria of 1% (dose difference)/1 mm (DTA) (C1), 2%/2 mm (C2), and 3%/3 mm (C3) were used to determine the percentage of points passed for each verification plan. For the MapCHECK 2 verification, the C3 criteria according to AAPM Task Group Report 119^(3)^ can be assumed as 4%/3 mm when an inherent 1% dosimetric uncertainty is added. According to the manufacturer, the 1% inherent dosimetric uncertainty includes differences between the absolute calibration of the MapCHECK and the standard dose value due to setup error, temperature change, accelerator output fluctuation, array calibration accuracy, and electronic measurement precision.

Absolute dose comparisons with 10% thresholds were adopted for all of the QA analyses. The 10% threshold excluded all points from being analyzed by the software that were below 10% of a selected point dose. A two‐tailed Student's *t*‐test was used for statistical analysis.^(11)^ The p‐values<0.05 were considered to be statistically significant at the 95% confidence level. In essence, three sets of comparisons were performed: PGAC vs. SGAC, SGAC vs. FBF, and PGAC vs. FBF. For FBF measurements, an overall passing rate for each plan was an MU‐weighted average of passing rates for individual fields.

### D. Effect of couch attenuation

**Figure 2:**
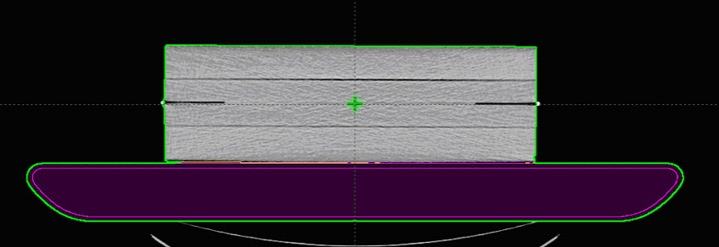
Modeling of the Varian couch top with 10 cm height of solid water blocks with isocenter placed at 5 cm depth in the Eclipse treatment planning system.

To inspect the effect of the couch attenuation, three possible clinical situations were studied using the PTW ionization chamber dose measurements. The QA verification plans were generated with and without the Varian couch top which consists of a low‐density foam core sandwiched with 4 mm carbon fiber plates. It is 53 cm wide, 230 cm long, and has varying thicknesses ranging from 5 cm (superior; gantry side) to 7.5 cm (inferior). The couch top was scanned to determine Hounsfield units (HUs). The HUs were determined to be −570(mean)±55(SD) for the carbon fiber and −959±1 for the foam core. The couch top (5 cm thick) where the phantom blocks were placed, as shown in Fig. 2, was modeled in the TPS. The ionization chamber measurements were also accomplished with the Varian couch top and the extended grid insert. The QA plans without the couch top were compared with the corresponding measurements with the original Varian couch top (P‐WOC (without couch) vs. M‐WC (with couch)) and the grid insert (P‐WOC vs. M‐WOC) by assuming attenuation is negligible through the insert, as shown in Fig. 1. In addition, the QA plans with the couch top were compared with measurements with the original Varian couch top (P‐WC vs. M‐WC). The effect of photon attenuation through the couch top for the IMRT QA measurements was further investigated by comparing the PGAC measurements with the Varian couch top (referred as PGAC) to those with the grid insert (referred as PGAC‐WOC) using the SNC MapCHECK 2, as shown in Fig. 1.

### E. Angular dependency of the diode detectors

In order to fully understand the PGAC, angular dependency of the MapCHECK 2 diode needed to be addressed. The angular dependency was investigated by comparing measurements with the extended grid couch top with the TPS calculations without couch top, for every 10°. With this setup, angular dependencies were studied for two different energies (6 MV and 10 MV) and field sizes (5cm×5cm and 10cm×10cm). All diode measurement points within a central area of 2cm×2cm (14 points) on the measurement plane around the isocenter were compared with the corresponding points from the TPS calculations for each angle. The mean dose difference ((M−P)/P×100%) of the 14 comparison points was computed.

**Figure 3:**
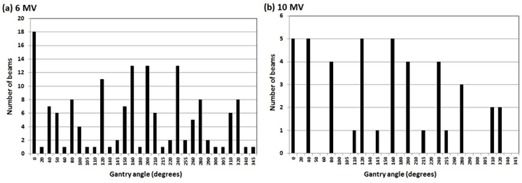
Number of beams at certain gantry angles used for PG‐FBF analysis for (a) 6 MV and (b) 10 MV. Split beams were individually counted in the number of beams.

In addition, the angular dependency of the IMRT QA measurements was further examined by FBF of actual patient gantry angle planned without the couch top and measured with the extended grid couch top (referred as PG‐FBF) and was compared with the original FBF (gantry angle=0°) using the *t*‐test. A total of 195 beams (152 for 6 MV and 43 for 10 MV) were compared based on gantry angle (one beam was excluded from the analysis due to insufficient dose points detected). The distribution of the gantry angles is shown in Fig. 3. For each gantry angle, an MU‐weighted average and SD of the γ passing rates for C1, C2, and C3 criteria were calculated. For some beam angles, there was only one beam used. In this instance, a SD could not be calculated.

## III. RESULTS

The verification of IMRT QA using the ionization chamber showed that our IMRT delivery system had good agreement between measurements and TPS calculations. The average percent differences of the chamber measurements were 0.4%±1.7% for H&N and 0.1%±1.5% for prostate which were <3% as proposed in the AAPM Task Group 119 report.^(3)^

### A. Compatibility of three different IMRT QA methods

The average γ passing rates of ten H&N and ten prostate verification plans were determined for all three γ criteria (C1, C2, and C3) and all three IMRT QA techniques, as shown in Table 2. The results (H&N/prostate) were 98.8%/98.9% (PGAC), 99.6%/100.0% (SGAC), and 99.5%/100.0% (FBF) with the C3 criteria. All techniques were determined clinically acceptable, with more than 95% of the points passing C3 criteria.^(4)^ Interestingly, PGAC showed lower average passing rates than SGAC and FBF for all cases and criteria. The p‐values of the *t*‐test comparing three different techniques are shown in Table 3. The two techniques, SGAC versus FBF, did not show statistically significant differences in their γ passing rates for all γ criteria in both H&N and prostate plans. However, the differences in the γ passing rates of PGAC compared to SGAC or FBF were not always statistically insignificant. It was statistically insignificant at the stricter γ criteria of C1 for the prostate plans (0.06 for PGAC vs. SGAC and 0.10 for PGAC vs. FBF), while it was statistically insignificant at the looser criteria of C3 for H&N plans (0.06 for PGAC vs. SGAC and 0.09 for PGAC vs. FBF). Their p‐values (PGAC vs. SGAC or FBF) were overall less than or close to 0.05 and much lower than those of SGAC vs. FBF, indicating higher probability of statistically significant differences.

The p‐values of comparisons between H&N and prostate plans are shown in Table 4. For PGAC, the differences of the γ passing rates between H&N and prostate plans were statistically insignificant for all criteria, indicating that the γ passing rates of PGAC was not highly affected by the level of intensity modulation. At the stricter YΓ criteria of C1, all techniques had statistically insignificant differences in the γ passing rates between H&N and prostate plans.

**Table 2:** Average passing rates of the γ test for the different IMRT QA techniques with different criteria

*Dose Diff‐DTA*		*Head and Neck*		*Prostate*	
*Criteria*	*QA Method*	*Mean*	*SD*	*Mean*	*SD*
C1	PGAC	56.1	8.6	58.7	8.5
(1%‐1 mm)	SGAC	70.9	8.3	68.3	8.1
	FBF	71.1	6.6	66.5	7.2
	PGAC‐WOC	75.1	7.8	69.7	12.2
	PG‐FBF	51.5	4.8	49.1	2.4
C2	PGAC	90.7	4.8	91.0	3.7
(2%‐2 mm)	SGAC	94.7	3.0	97.9	3.5
	FBF	95.0	1.9	97.2	1.9
	PGAC‐WOC	96.9	2.4	95.9	3.1
	PG‐FBF	82.0	3.8	75.8	3.5
C3	PGAC	98.8	1.1	98.9	0.6
(3%‐3 mm)	SGAC	99.6	0.4	100.0	0.0
	FBF	99.5	0.3	100.0	0.1
	PGAC‐WOC	99.6	0.6	99.7	0.5
	PG‐FBF	93.3	2.3	87.5	3.9

**Table 3:** The p‐values of comparison among the MapCHECK QAs

	*Cl (1%/1 mm)*			*C2 (2%/2 mm)*			*C3 (3%/3 mm)*		
	*PGAC vs. SGAC*	*SGAC vs. FBF*	*PGAC vs. FBF*	*PGAC vs. SGAC*	*SGAC vs. FBF*	*PGAC vs. FBF*	*PGAC vs. SGAC*	*SGAC vs. FBF*	*PGAC vs. FBF*
H&N	0.01	0.91	<0.004	0.08	0.63	0.03	0.06	0.44	0.09
Prostate	0.06	0.52	0.10	0.00	0.63	<0.0001	<0.0001	0.34	<0.0001
*PGAC‐WOC vs. PGAC*	*PGAC‐WOC vs. SGAC*	*PGAC‐WOC vs. FBF*	*PGAC‐WOC vs. PGAC*	*PGAC‐WOC vs. SGAC*	*PGAC‐WOC vs. FBF*	*PGAC‐WOC vs. PGAC*	*PGAC‐WOC vs. SGAC*	*PGAC‐WOC vs. FBF*
H&N	<0.0002	0.20	0.24	0.01	0.09	0.06	0.10	0.87	0.77
Prostate	<0.002	0.81	0.58	<0.0005	0.20	0.18	<0.0001	0.06	0.08

**Table 4:** The p‐values of IMRT QA between H&N and prostate plans

*Dose Diff–DTA Criteria*	*PGAC*	*SGAC*	*FBF*	*PGAC‐WOC*
C1 (1%/1 mm)	0.46	0.31	0.24	0.34
C2 (2%/2 mm)	0.80	0.02	0.02	0.54
C3 (3%/3 mm)	0.72	0.02	<0.003	0.79

For the looser γ criteria of C2 and C3, statistically significant differences were observed for the SGAC and FBF techniques, indicating that the γ passing rates of these techniques are more likely dependent on the level of intensity modulation.

### B. Effect of couch attenuation

The average percent differences between planning (P) and measurement (M) using the ionization chamber for three different situations are shown in Table 5: P and M with the Varian couch (S1: P‐WC vs. M‐WC), P without couch and M with the Varian couch (S2: P‐WOC vs. M‐WC), and P without couch and M with the grid extension (S3: P‐WOC vs. M‐WOC). Note that S3 was the verification of the IMRT QA delivery. Comparisons using the *t*‐test are also given for the three different situations. The average percent differences of all 20 QA measurements (tenH&N+ten prostate cases)forS1(range:−0.5%~3.2%),S2(−1.7%~1.6%),andS3(−2.3%~4.1%)were1.3%±1.0%,−0.2%±1.0%,and0.3%±1.5%, respectively. Statistically insignificant differences were observed for S2 vs. S3 in all comparisons. The couch modeling in the Varian TPS (S1) made the chamber measurements higher than planned doses in 19 out of 20 IMRT QAs. In most of the compared cases, S1 showed statistically significant differences with S2 and S3 (except for S1 vs. S3 for H&N); however, the absolute differences of the average dose differences were less than 2.0%.

The average γ passing rates of PGAC‐WOC using MapCHECK 2 are shown in Table 2, which were comparable to those of SGAC and FBF but higher than those of PGAC for all plans and criteria. Table 3 shows comparisons between PGAC‐WOC and the three QA techniques (PGAC, SGAC, and FBF). In all cases (except C3 of H&N), PGAC‐WOC showed statistically significant differences with PGAC. The differences in the γ passing rates between PGAC‐WOC and SGAC or FBF were statistically insignificant in all cases.

**Table 5:** Average dose difference of ionization chamber measurements for three different situations concerning the couch

	*S1 (P‐WC vs. M‐WC)*	*S2 (P‐WOC vs. M‐WC)*	*S3 (P‐WOC vs. M‐WOC)*
H&N	1.0%±0.7%	−0.4%±0.9%	0.4%±1.7%
Prostate	1.7%±1.1%	0.0%±1.0%	0.1%±1.5%
Overall	1.3%±1.0%	−0.2%±1.0%	0.3%±1.5%
*p‐value (t‐test)*	*S1 vs. S2*	*S2 vs. S3*	*S1 vs. S3*
H&N	<0.00001	0.20	0.36
Prostate	<0.00001	0.78	0.03
Overall	<0.00001	0.23	0.02

### C. Angular dependency of diode detectors

**Figure 4:**
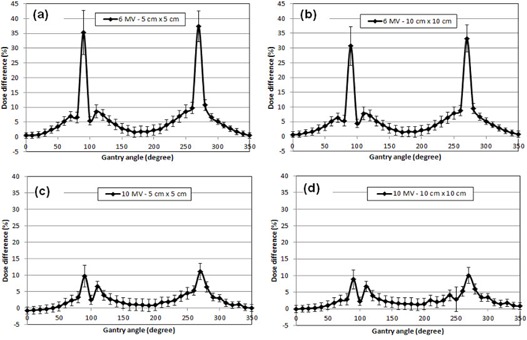
The percent dose difference between measurement using MapCHECK 2 and planning (all measurement and planning points within a 2cm×2cm area were averaged and compared) at different gantry angles for different energies (6 MV and 10 MV) and field sizes (5cm×5cm and 10cm×10cm).

**Figure 5:**
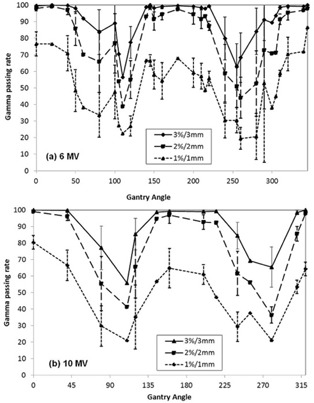
The average γ passing rates for each beam angle delivered using PG‐FBF technique for 6 MV and 10 MV beams.

Figure 4 shows the percent dose differences between measurement and planning for a gantry angle at a certain field size and energy. There was a huge angular dependency of the MapCHECK 2 diode detectors around the gantry angles of 90° and 270°. It should be noted that these angles were not used for planning, as shown in Fig. 3. The percent dose differences were as large as 37% for the 6 MV photon beam using a 5cm×5cm field at an angle of 270°. This is similar to other studies who found errors as large as 25%.^(12)^ In a majority of the beam angles, the measured dose was higher than the planned dose. The dose difference between planning and measurement was much higher for 6 MV than 10 MV. The maximum percent dose differences for 6 MV and 10 MV were 37% and 13%, respectively. For the 6 MV beams, the 5cm×5cm field had a larger dose difference than the 10cm×10cm field near the angles of 90° and 270°. The ten MV beams exhibited the same trend as the 6 MV, but the dependence on field size was much smaller than it was with 6 MV beams. Figure 5 shows the average γ passing rates of PG‐FBF based on the gantry angles. The gantry angles which had the lowest γ passing rates occurred around 110° and 280° for both energies, which nearly corresponded with the gantry angles with the largest dose difference seen in Fig. 4. The overall average γ passing rate of PG‐FBF was significantly lower than those of FBF for all γ criteria, as shown in Table 2.

## IV. DISCUSSION

SGAC and FBF showed statistically insignificant differences in γ passing rates for both H&N and prostate and for all γ criteria, while PGAC compared to the other two techniques in most cases showed statistically significant differences in γ passing rates (Table 3). This distinction may result from these four reasons: (1) delivery techniques, (2) treatment couch attenuation, (3) angular dependency of the MapCHECK 2 device, and (4) the γ test itself. Since PGAC uses the actual treatment angles, it inherently tests for problems such as gantry sag and possible changes in the motion of the MLCs due to gravity at different gantry angles, while FBF and SGAC do not. The treatment couch may also play a small role in making the passing rates for PGAC different from SGAC. A significant difference arises between delivering a beam at actual gantry angles and delivering the beam orthogonal to the surface of the MapCHECK 2 due to the angular dependency of the diodes, especially at gantry angles of 90° and 270°. The properties of the γ test also play a role in the different passing rates between PGAC and FBF. The goal of IMRT is to deliver nonuniform fields which will in summation lead to a uniform field in a target. For FBF, each field is made mostly of high gradient regions, while composite plans have a more uniform distribution. The high gradients measured for FBF (or SGAC) make the γ test more dependent on DTA, while the uniform distribution in PGAC makes the γ test more dependent on dose difference. SGAC and FBF are comparable because they both deliver their beams at gantry angle of zero which makes the passing rates free from couch attenuation, angular dependency issues, gantry sag, and gravity effects on MLC motion.

The goal of the comparison between H&N and prostate was to determine whether these different IMRT QA techniques could distinguish different degrees of intensity modulation (Table 4). PGAC and PGAC‐WOC showed that these techniques do not significantly rely on the degree of modulation, while SGAC, FBF, and PG‐FBF are able to distinguish between differences in modulation but only at the γ criteria of C2 and C3. These results are similar to a recent study,^(5)^ which showed statistically significant differences between H&N and prostate for C3 criteria using FBF. However, it is hard to determine which method is more clinically meaningful. PGAC is more dependent on dose difference rather than DTA and simulates actual treatment conditions better, which makes this technique the most clinically meaningful, while it substantially suffers from angular dependency. SGAC has known issues of masking errors upon summation, and FBF technique has been shown insufficient at detecting clinically relevant dose errors.^(13)^ However, there are no studies to indicate that PGAC would be a better option at detecting clinically relevant dose errors.

Even if this study was performed with the step‐and‐shoot (SS) IMRT plans, we believe that similar results would be obtained with sliding window (SW) IMRT delivery using the Eclipse TPS. In the Eclipse IMRT planning, the same leaf‐sequencing algorithm is used to convert optimized intensity maps into deliverable aperture shapes for both SS and SW deliveries with a smaller number of segments sampled for the SS IMRT delivery. Considering the small active detector area (0.8mm×0.8mm) and high detectable dose rate (up to 18 cGy/s) of MapCHECK 2, any significant difference between SS and SW IMRT deliveries in IMRT QA will not be observed. However, different leaf‐sequencing algorithms which can make numerous small fields for SW IMRT delivery may not present the same QA outcomes. This requires further research.

Results from our ionization chamber measurements indicated that there was an almost statistically negligible dose difference (0.5%) between measurements with couch and without couch (S2 vs. S3 in Table 5). It should be noted that this result was only valid for the specific Varian IGRT couch top. In contrast, there was a statistically significant dose difference observed between the ionization chamber measurement and the plan dose when the treatment couch was modeled in the TPS. This is probably due to difficulties of modeling the treatment couch in a TPS.^(14)^ Modeling of the treatment couch in the TPS for use in patient plans is essential for many couch tops, especially those with rails, to ensure the patient is not being underdosed due to the attenuation of the table. In one study, it was shown that excluding the couch and rails lowered the tumor control probability (TCP) by 6.3% on average, rendering the treatment plan clinically unacceptable.^(10)^ The accuracy of planning with a couch top strongly depends on the determination of HU value of the couch top. Even if the actual scanned data were used in this study, the standard deviation of the average HU value was relatively large due to blurring of thin couch top structure on a CT image. The accuracy of determining the HU value can be further improved by an iterative measurement method illustrated by Njeh et al.^(15)^ In addition, PGAC was not comparable with SGAC in most cases, while PGAC‐WOC was highly comparable to SGAC (Table 2) and difference in γ passing rates between PGAC and PGAC‐WOC was statistically significant (Table 3). This indicates that the couch attenuation can affect γ passing rates. However, it should be noted that the QA results with the ionization chamber were clinically acceptable (average dose difference <3%) in all cases and both PGAC and PGAC‐WOC passed the standard IMRT QA of 95% of points passing the C3 criteria.

The angular dependence of the diodes in the MapCHECK 2 device is due to its construction which consists of mounting a diode on a circuit board with a copper plate.^(16)^ In the paper, the researcher states: “Low energy electrons are backscattered at the interface of low and high atomic number materials and this results in higher diode sensitivity to photons that enter from the directions of the front and back surfaces of the copper plate.”^(16)^ This explains the differences in responses observed with energy of the beam. Lower energy beams have higher backscatter, which is why the 6 MV beams overrespond much more than the 10 MV beams.

Calibration of the MapCHECK 2 device is responsible for the smaller field sizes having a higher angular dependency. The MapCHECK 2 is calibrated by delivering a known amount of dose using a 10cm×10cm field.^(17)^ Larger field sizes have an increased scatter component which leads to higher dose. Dose by fields smaller than 10cm×10cm will be overestimated because of the increased scatter measured in the calibrated 10cm×10cm field (1% underreading of the MapCHECK 2 array for 2cm×2cm static field). The angular dependency issue is also a problem in other planar array detectors such as the IBA MatriXX ionization chamber array where up to an 8% difference in detector response has been observed.^(18)^ The difference between these two devices is that the MatriXX array tends to underrespond at most angles, while MapCHECK 2 tends to overrespond at most angles. Some groups^(^ ^12^ ^,^ ^19^ ^)^ have tried to reduce or eliminate the angular dependency found in MapCHECK 2. The angular dependency of the MapCHECK 2 is shown to be largest at angles of near 90° and 270°. If 90° and 270° beams are used, it is highly recommended that sagittal orientation (detector array vertical to the couch top) be used to set up the MapCHECK 2.

An interesting result was that the γ passing rates for 10 MV were similar to those for 6 MV even though 6 MV beams had a much higher angular dependence. Another interesting result was the fact that high γ passing rates were observed in PGAC, while PG‐FBF failed in most cases. These observations may be due to masking errors in PGAC with some fields underdosing and other fields overdosing and dose comparison by a normalization point dose in the γ test. The γ test uses dose difference between measurements and plans normalized to a selected point (usually the maximum dose level) to determine whether a comparison point passes the test. Composite dose distributions such as PGAC or SGAC have much higher dose for the normalization point than a single field measurement of PG‐FBF, and thus a probability of passing the γ test is higher for certain points whose dose contribution comes from a single field. Another issue with PG‐FBF is that, for some beams, part of the field is not sampled due to the dimension of the detector. Having a small sample of points will lower the yγ passing rate even if only a few points fail.

There are a few limitations of this research that need to be discussed. The prostate plans were all similar in complexity, while the H&N plans varied in complexity and site. Another limitation is the sample size of patients studied. Having more patients would give a more reliable answer to the comparability of these techniques. For the PG‐FBF study, none of our patients had beam angles of 90° and 270°. These are the two most important angles that were needed for studying angular dependency because these angles have the highest overresponse. And for some beam angles only one measurement was implemented. This study also could have been improved if PG‐FBF was performed using a mounting fixture. This would allow us to study differences in passing rates with gantry sag and gravity effects of the MLCs taken into account.

## V. CONCLUSIONS

Our results show that in most cases PGAC (planning without couch and measurement with couch) has statistically significant difference with SGAC or FBF, while PGAC‐WOC (planning and measurement without couch) has statistically insignificant difference with SGAC or FBF. In addition, FBF and SGAC were found to be comparable in all cases. Our study indicates that the treatment couch has a significant impact on γ passing rates, but may not cause clinically significant dose errors in IMRT QA especially when using an ionization chamber. This study shows that the MapCHECK 2 device has large angular dependency, especially at gantry angles of 90° and 270°, which can dramatically affect the γ passing rate. The PGAC technique could potentially be a clinically superior IMRT QA technique due to simulation of actual treatment conditions, as long as angular dependency issues are taken into account. With proper consideration of couch attenuation and beam arrangement, the MapCHECK 2 will produce clinically comparable QA results using the three different planar IMRT QA techniques.
